# Comparison of Jugular vs. Saphenous Blood Samples, Intrarater and In-Between Device Reliability of Clinically Used ROTEM S Parameters in Dogs

**DOI:** 10.3390/ani12162101

**Published:** 2022-08-17

**Authors:** Johanna Vuille-dit-Bille, Nicole Weingand, Rahel Jud Schefer, Martina Stirn, Katja-Nicole Adamik, Justus M. K. Rathmann, Nadja E. Sigrist

**Affiliations:** 1 Division of Small Animal Emergency and Critical Care, Department of Veterinary Clinical Medicine, Vetsuisse Faculty, University of Bern, 3012 Bern, Switzerland; 2 Department for Small Animals, Vetsuisse Faculty, University of Zurich, 8057 Zurich, Switzerland; 3 Department for Clinical Diagnostics and Services, Vetsuisse Faculty, University of Zurich, 8057 Zurich, Switzerland; 4 Institute of Sociology, University of Zurich, 8050 Zurich, Switzerland

**Keywords:** ROTEM, canine, sampling site, coagulation, coefficient of variation, thromboelastometry

## Abstract

**Simple Summary:**

Viscoelastic coagulation tests such as rotational Thromboelastometry (ROTEM) have many theoretical advantages compared to traditional coagulation testing. As a point-of-care diagnostic device, ROTEM results are directly part of treatment decisions. Therefore, it is crucial to know its reliability and precision. Most recommendations for ROTEM S analyses originate from Thromboelastography (TEG), another viscoelastic coagulation assay. However, evidence about how preanalytical and analytical factors, such as sample collection technique, sample handling and the analysis itself, influence ROTEM results is scarce. Due to the absence of a gold standard method, we assessed accuracy with the coefficient of variation and intraclass correlation coefficient and examined the influence of blood collection site, as well as intrarater and in-between device variability, on ROTEM S results of clinically healthy dogs. We found significant changes between ROTEM S parameters from different blood collection sites and significant intrarater and in-between device variability. These findings were most prominent in tissue-factor-activated tests. To ensure patient safety, we therefore suggest running duplicate measurements and to interpret results obtained from tissue-factor-activated tests with caution, since some of their coefficients of variation were moderate to high.

**Abstract:**

Rotational Thromboelastometry (ROTEM) allows for the global assessment of hemostasis in whole blood samples. Preanalytical and analytical factors may influence test results, and data about the reliability and reproducibility of lyophilized ROTEM tests are scarce. Therefore, the objective of this study was to evaluate the influence of blood collection site on ROTEM S parameters and to assess intrarater and in-between device variability. A total of thirty, healthy, staff-owned dogs were included. Blood collection and ROTEM analysis were performed by trained staff according to a standardized protocol. Extrinsically activated (tissue factor; Ex-TEM S), with the addition of cytochalasin for platelet inhibition (Fib-TEM S), and intrinsically activated (In-TEM) analyses were performed. Analysis of our data showed significant variability for various Ex-TEM S and Fib-TEM S parameters from different collection sites and intrarater and in-between device measurements. We conclude that serial monitoring with ROTEM should be performed on the same device, with blood always taken from the same collection site using a standardized blood sampling technique. While In-TEM S, apart from maximum lysis, showed very stable and reliable results, we suggest interpreting especially clotting and clot formation parameters from Ex-TEM S and Fib-TEM S tests with caution and using duplicate measurements to detect outliers and to prevent initiation of incorrect therapies.

## 1. Introduction

The methodology of viscoelastic coagulation assays such as rotational Thromboelastometry (ROTEM) or Thromboelastography (TEG) was first described by Hartert more than 50 years ago [1]. Both ROTEM and TEG are point-of-care (POC) devices that allow for a global assessment of hemostasis in whole blood samples and therefore provide information about the activation of the coagulation cascade, the kinetics of clot formation, and the mechanical properties of the clot and clot dissolution [2,3]. The utilization of whole blood provides charged phospholipid cell surfaces for enzymatic reactions and platelets, which allows for an environment that more likely reflects hemostatic processes as they occur in vivo [4,5].

In veterinary medicine, TEG/ROTEM have been validated for use in horses [6,7], dogs [8,9] and cats [10,11]. Although, theoretically, there are many advantages of viscoelastic tests compared to traditional coagulation testing in plasma, it is important to note that multiple preanalytical and analytical factors can influence test results [4,12,13], including sample collection technique (size of chosen vessel, amount of vessel occlusion, venipuncture technique, use of vacutainers, use of syringes), sample handling (storage time before initiation of the test, storage temperature), and the analysis itself (variability of ROTEM devices, operator variability).

In 2014, the “Partnership on Rotational ViscoElastic Test Standardization” (PROVETS) provided guidelines for veterinary use of TEG and ROTEM to improve assay consistency and result interpretation and to allow for the comparison of results between different testing centers and enable further progress in this field [13,14].

Due to the scarcity of ROTEM studies at this timepoint, most preanalytical recommendations for ROTEM analysis originate from TEG studies. Previous publications from human and veterinary medicine have shown that TEG and ROTEM provide similar but not interchangeable results [15,16], that they have different intra- and inter-assay variability [17,18] and that variation between assays, locations and devices may be very high [18].

Smith et al. found that generally good interduplicate coefficients of variance below 20% for Ex-TEM (tissue factor-activated temogram) initiated coagulation, using liquid test reagents provided by the ROTEM manufacturer [12]. To our knowledge, no studies have evaluated intrarater, interrater and inter-assay variability in canine blood samples undergoing ROTEM S analysis. ROTEM S reagents contain lyophilized activation factors, which is in contrast to liquid ROTEM reagents, which require an additional step of recalcification of blood samples prior to analysis.

To the author’s knowledge, there is also no study evaluating the impact of blood collection site for ROTEM analysis in dogs. In cats, jugular venous blood samples analyzed with VCM Vet, another viscoelastic POC device, appear to be more hypercoagulable compared to those taken from the medial saphenous vein [19].

The objective of our prospective observational study was three-fold: to (a) evaluate the impact of blood collection site and technique on viscoelastic test results, to (b) assess the intra- and interrater variability of trained staff using ROTEM delta and to (c) compare ROTEM parameters of the same blood sample that were analyzed on two different ROTEM delta devices. Our null hypothesis was that parameters do not differ between two samples.

## 2. Materials and Methods

Healthy, adult, staff-owned dogs were recruited for the purposes of this and another study [20]. The study was approved by the ethics committee on animal research of the Canton of Zurich (ZH 057/19) and signed owner consent was available. Dogs eligible for this study needed to be considered healthy based on complete history and physical examination and have no history of a coagulation disorder.

Dogs weighing <2 kg and dogs showing any signs of stress or struggling during blood collection were excluded. Breed, age, gender and body weight was recorded for every dog.

To minimize preanalytical errors, blood collection and analyses were performed according to an institutional standard protocol based on manufacturer’s instructions and international guidelines [13]. Care was taken that minimum vessel occlusion was used, venipuncture was performed atraumatically and that there was a constant blood flow into the syringe or tube. A discard sample was not used, but samples were only used for analysis if blood collection was considered atraumatic.

Venipuncture was performed by two operators (J.V. and N.W.). For jugular vein sampling, a 22 G needle was connected to a 5 mL syringe and 4 mL of blood was drawn using minimal vessel occlusion and mild aspiration with a vacuum of 1 mL inside the syringe. After removal of the needle, aliquot portions of the blood were immediately transferred into 2 or 3 3.8% sodium citrate tubes (Microtube 1.3 mL 9 NC, Sarstedt AG, Nürnbrecht, Germany), resulting in a 1:9 ratio of citrate with blood. With the minimum possible delay, a second sample was drawn from some of the dogs (weighing > 10 kg, being cooperative and pending owner consent) from the lateral saphenous vein by the same veterinarian. Again, a 22 G hypodermic needle was used but the blood was allowed to flow directly into 2 or 3 1.3 mL 3.8% sodium citrate tubes. Each tube was inverted carefully several times. The citrated blood was kept at 37 °C, using the analyzer’s designated warming plate or a 37 °C heating chamber, until the tests were run.

Rotational thromboelastometry analyses (ROTEM Delta, TEM Innovations GmbH, Munich, Germany) were performed by the same three experienced veterinarians.

Tests performed included Ex-TEM S (extrinsic activation by tissue factor), In-TEM S (ellagic acid activation) and Fib-TEM S (tissue factor activation with cytochalasin D added to block platelets). They were performed as previously described [9] on two ROTEM delta devices. Apart from Fib-TEM S, which was run for only 20 min, all samples were analyzed for 60 min at 37 °C.

For the evaluation of the impact of blood collection site on viscoelastic test results, Ex-TEM S, In-TEM S and Fib-TEM S analyses were performed from both jugular and saphenous blood samples at the same time point after blood sampling (10, 30 or 70 min) on the same ROTEM device by the same operator.

For assessment of the intrarater variability of trained staff using ROTEM delta, some of the samples were run in duplicates on the same ROTEM device and by the same operator. Based on the availability of free channels, some samples were additionally simultaneously run on two different ROTEM delta devices (again by the same operator) to assess in-between device variability.

All ROTEM tracings were visually evaluated for artefacts by the three operators (JV, NW, NS). If an artefact was suspected, the abnormal parameter or the temogram was excluded.

The following parameters were extracted from the ROTEM database and copied into a spreadsheet: clotting time (CT), clot formation time (CFT), maximum clot firmness (MCF), alpha-angle (α), maximum lysis (ML), maximum clot elasticity (MCE) and G, a calculated measure of total clot strength (G = 5000 × MCF/(100−MCF)). If in any profile MCF did not reach 20 mm, an infinite CFT was defined as 3600 s. For Fib-TEM S, only CT, MCF, MCE and G were analyzed. A green line in the Fib-TEM tracing was defined as a Fib-TEM-MCF of 1 mm.

Tracings were categorized as hypo-, normo- or hypercoagulable based on G compared to the reference interval of G [9].

### Statistical Analysis

The data were manually transferred from the ROTEM database into an Excel spreadsheet. Afterwards, they were recoded and analyzed with the data analysis programs R (R Core Team, 2020; version 3.9.1) and RStudio (RStudio Team, 2020; version 1.2.1335). Quantile plots were used to verify that the data were normally distributed. The analyzed variables were for the largely normally distributed. However, there were very few deviations at the tails of the distribution, but this is not surprising given the small sample size. Differences between measurements of the parameter were depicted based on the coefficient of variation (CoV), which was calculated by dividing the standard deviation by the mean times 100. The statistical significance of the differences between the respective first and second measurement was estimated using paired *t*-tests. Following the approach by Junge et al. [7], we classified the CoV as follows: <5%, very low variability; 5–15%, low variability; 15–25%, moderate variability; >25%, high variability. Additionally, the interclass correlation coefficients to estimate the reliability of the measurements was calculated. Koo and Li were used as a guideline for interpreting the coefficients: values <0.5 are indicative of poor reliability, values between 0.5 and 0.75 indicate moderate reliability, values between 0.75 and 0.9 indicate good reliability, and values greater than 0.90 indicate excellent reliability [21]. Following Bartko [22], negative ICC values are reported as 0.

For the intrarater variability, an ordinary least squares regression model to test whether the person performing the blood sampling or the time of blood sampling had an influence on the repeated measurements was performed. The z-standardized difference between the respective first and second measurements was used as the dependent variable. Person and timing were the dependent variables.

## 3. Results

### 3.1. Population

A total of 30 dogs were included. Eighteen dogs (60%) were males (7 intact, 11 neutered) and twelve dogs (40%) were female (2 intact, 10 neutered). The median age of the dogs was 75.5 months (range, 13–146 months) and the median body weight was 22.9 kg (range, 2.3–59 kg). Fifteen dogs were crossbreed, while the most common other breeds were Labrador Retriever (*n* = 3), Chihuahua (*n* = 2), German Wirehaired Pointer (*n* = 2) and American Pitbull (*n* = 2). The remaining dogs (*n* = 6) belonged to various other breeds, with one individuum per breed.

### 3.2. Comparison of Jugular and Saphenous Blood Samples

Thirty-six Ex-TEM S, 18 In-TEM S and 19 Fib-TEM S measurements of both jugular and saphenous blood were compared. Results assessing the comparison of jugular and saphenous ROTEM measurements are presented in Table 1.

All parameters, with the exemption of Ex-TEM CFT, In-TEM CT and ML and FibTEM CT, MCE and G, were significantly different between the two sampling sites.

Despite the statistically significant differences, except for Ex-TEM CFT sampled from the jugular and Fib-TEM CT sampled from the saphenous vein, all results were within the reference intervals.

The coefficient of variation was low, except for Ex-TEM CT, CFT, ML, In-TEM ML and all investigated Fib-TEM parameters. Intraclass correlation coefficient was good to excellent in In-TEM CFT, alpha angle, MCF, MCE, G and Fib-TEM CT, while ICC was poor for Ex-TEM CT, CFT, ML, IN-TEM CT and ML and Fib-TEM MCF, MCE and G.

All Ex-TEM parameters sampled from the saphenous vein were more hypercoagulable compared to their matched sample from the jugular vein. This trend could not be observed in In-TEM or Fib-TEM parameters. The different sampling sites resulted in a change of coagulation status in 3/36 Ex-TEM (1 hypo- to normocoagulable, 2 normo- to hypercoagulable), 1/18 In-TEM (normo- to hypercoagulable) and 1/19 Fib-TEM (normo- to hypercoagulable) measurements (Table 2).

### 3.3. Intrarater Variability

Twenty-three duplicate Ex-TEM S measurements, 18 In-TEM S and 21 Fib-TEM S were included. Duplicates were analyzed on the same device. Results of ROTEM measurements assessing intrarater variability are shown in Table 3. Coefficient of variation (CoV) was considered low (13%) for Ex-TEM alpha and MCF, moderate (19%) for Ex-TEM MCE and G and high for all other Ex-TEM parameters ranging up to unacceptably high values of 40%. Except for both measurements of CFT, all results were within the reference intervals [9]. Within Ex-TEM results, a change in coagulation status (based on G) between the repeated measurements was noted in 7/23 cases (3x normo- to hypocoagulable, 3x hypo- to normocoagulable, 1x normo- to hypercoagulable) (Table 2). Intraclass correlation coefficient was considered good in Ex-TEM MCE, G and ML and only moderate in Ex-TEM MCF.

The performance of the In-TEM test showed excellent CoVs for CT (5%), alpha (4%), MCF (1%), MCE (3%) and G (3%). The intraclass correlation coefficient was considered excellent or good for all In-TEM parameters except ML. All In-TEM measurements were within the reference intervals and there was no change in coagulation status (Table 2).

Fib-TEM MCF, MCE and G showed low variability with CoVs of 12%, 12% and 13%, respectively, while ICC was considered good for clot strength parameters. Apart from one measurement of CT, all results were within the reference range. A change of coagulation status was detected in 1/21 cases (normo- to hypercoagulable) (Table 2).

A linear regression model showed that there was no significant influence on CoV by the operator running the tests or by the timepoint after blood sampling (10, 30 or 70 min) tests were run, while the Ex-TEM test was found to be a significant confounding factor. Compared to an Ex-TEM measurement, the difference between the first and second measurement is 0.74 and 0.67 standard deviations smaller for a Fib-TEM and an In-TEM test, respectively. This means that repeated measurements by both Fib-TEM and In-TEM tests are considerably more reliable than an Ex-TEM test.

### 3.4. Inter-Device Variability

For Ex-TEM, we ran seven duplicate measurements on device 1 comparing the mean values with the mean value of seven duplicate measurements of the same blood sample tested with device 2. The same procedure was done for six duplicates In-TEM and four duplicate Fib-TEM tests. The results of all measurements investigating the in-between device variability are shown in Table 4.

Inter-device variability was considered low only for Ex-TEM MCF and all In-TEM parameters except ML, while ICC was good to excellent in Ex-TEM MCE, G, IN-TEM CT, CFT and alpha angle. Variability and ICC were inacceptable in Ex-TEM CT, CFT, In-TEM ML and Fib-TEM CT (Table 4).

Coagulation status changed in 1/7 Ex-TEM (normo- to hypocoagulable), 1/4 Fib-TEM (normo- to hypocoagulable) and no In-TEM test (Table 2).

## 4. Discussion

This study investigating the effects of different blood sampling sites, intrarater and in-between device variability on ROTEM S parameters showed significant changes between ROTEM S parameters from different collection sites, duplicate measurements and in-between device measurements.

As a POC diagnostic device, ROTEM results are part of a patient’s treatment decisions. It is therefore crucial to know its reliability. The accuracy of a test is assessed by determining how closely the results match the gold standard. Reliability and precision can be investigated with a variability of assays such as CoV, intraclass correlation coefficient, Bland–Altman analysis and various correlation tests among others. Due to the lack of a gold standard method, the coefficient of variation and intraclass correlation were chosen for analysis in order to be able to compare results to previous publications [17,23].

Previous work about types and frequencies of general laboratory errors has highlighted the occurrence of mistakes primarily in the preanalytical period (61.9%) compared to the analytical (15%) or postanalytical period (23.1%) [24]. Several preanalytical and analytical factors are suspected to potentially influence ROTEM results in human [23] as well as in veterinary patients [6,12]. Proposed preanalytical factors include sampling site, analysis temperature and storage time.

Analysis of our data showed significant variability with different collection sites, intrarater and in-between device measurements as well as for specific tests and parameters.

### 4.1. Comparison of Jugular and Venous Blood Collection Site

Veterinary viscoelastic testing guidelines suggest that blood should be free-flowing and, in small animals, jugular venipuncture is preferable [14]. This recommendation is based on one study reporting small but significant differences in results with different blood collection methods and sites using TEG [25]. However, in clinical practice, blood collection from the saphenous vein may be preferable in animals that potentially are hypocoagulable or are too nervous for jugular blood sampling.

ROTEM S analysis comparing jugular and saphenous blood collection sites in our population of healthy dogs resulted in significant differences in several parameters, which is in accordance with a previous study using a viscoelastic method in cats [19] but not using TEG in dogs [25]. In-TEM parameters were most reliable in reproducibility, followed by Ex-TEM clot strength parameters while Fib-TEM parameters showed both a poor ICC and a high CoV. Walker et al. did not find significant differences between jugular and saphenous recalcified blood samples of dogs tested with TEG [25]. However, they found that blood samples taken from the jugular vein with syringe aspiration were hypercoagulable compared to the samples taken via evacuated tube from the saphenous vein. In cats, jugular non-anticoagulated whole blood samples analyzed with a viscoelastic test similar to ROTEM appear to be more hypercoagulable compared to those taken from the medial saphenous vein [19]. The more hypercoagulable samples from jugular blood are in contrast to our finding that all Ex-TEM parameters from the saphenous vein, flowing freely into the citrate tubes over a 22 G hypodermic needle, were more hypercoagulable compared to their matched samples from the jugular vein taken with a syringe; a trend that was less obvious but still present in In-TEM S or Fib-TEM S tests. We cannot exclude an influence of sampling method (syringe aspiration vs. free flowing) on our results. A previous study did not find a significant influence of sample collection techniques on TEG results [26]. Additionally, blood collection with free-flowing blood has a lower shear rate than blood collected with a syringe and vacuum, leading to less platelet activation [27,28]. According to this, a blood collection with free-flowing blood (blood collection from the saphenous vein) has a lower shear rate than blood collected with a syringe and vacuum (blood collection from the jugular vein); however, we obtained rather hypercoagulable results from the free-flowing saphenous blood samples. We therefore conclude that the smaller vascular lumen, leading to an increase in shear stress, shedding of procoagulant-containing microparticles and therefore intensified platelet activation, rather than the blood collection technique, is responsible for the different results between jugular and lateral saphenous blood samples.

### 4.2. Intrarater Variability

Previous viscoelastic studies in people and dogs report different intra- and interrater variability using duplicate analysis. Anderson et al. reported excellent intrarater coefficients of variation <10% for In-TEM S and Fib-TEM S in human blood but did not investigate Ex-TEM S [17]. Another study with human blood samples describes good reproducibility in Ex-TEM and In-TEM (liquid tests) with intra-device CoVs of <6% for CFT, alpha and MCF and 15% for CT [23]. Samples were run in duplicate on the same device; unfortunately, it is not possible to determine whether the samples were tested by the same operator or not. Mauch et al. published good intraclass correlation coefficients (ICC) for Ex-TEM, In-TEM and Fib-TEM MCF <5% and high (unacceptable) ICC for CT and CFT in piglets [29]. Overall, CT seems to have the highest variability in all studies, which was also a finding in Ex-TEM S and Fib-TEM S of our patient population. Our study results show mixed variability in Ex-TEM S parameters, with Ex-TEM alpha, MCF, MCE and G having low coefficients of variation. All Fib-TEM parameters had a low variability of <15% except for CT. The In-TEM S parameters were clearly more reliable. The variability results are supported by the additionally performed ICC, which showed good-to-excellent results for most parameters, with the exception of Ex-TEM CT and CFT and In-TEM ML.

Thirteen percent of duplicate samples showed a change in coagulation status when comparing the two measurements based on G. This has to be taken into account when interpreting results from single test results. A linear regression model revealed no significant influence by the operator running the tests, indicating that interrater variability was negligible. Nevertheless, it has to be taken into consideration that our three operators were experienced in performing ROTEM assays, while ROTEM may also be used by less trained personal leading to potentially higher variation.

### 4.3. Inter-Device Variability

Inter-device variability and ICC showed more variability than intrarater comparison, with Ex-TEM S as well as all Fib-TEM S parameters showing a moderate-to-high variability and poor-to-moderate ICC. Because of the small sample size, significance statements must be interpreted with caution, as distinction between causality and correlation was not reliably possible. A change of coagulation status was observed in 12% of measurements; therefore, current guidelines based on TEG studies recommending serial measurement of tests on the same device [13] seem to apply for ROTEM analysis as well.

Compared to previous studies investigating TEG or liquid ROTEM test results, specifically the Ex-TEM S and Fib-TEM S tests showed a great variety of values for intrarater and inter-device CoV. We can only speculate about the reasons for this discrepancy. The repeatability of assays may depend not only on pre-analytical factors, which have been eliminated as much as possible in our study setup, but also on the test reagent itself. Since Ex-TEM and Fib-TEM both contain tissue factor as activator, the activator may be responsible for our findings. As suggested before, the concentration of tissue factor in the test reagents may be insufficient for a rapid and adequate thrombin burst. An earlier study has shown that lyophilized Ex-TEM reagents lead to smaller temograms than the liquid Ex-TEM reagents, resulting in lower MCF reference intervals [9]. High activator concentrations leading to a rapid thrombin burst are expected to prevent any influence of sample handling on ROTEM results [12]. Although ROTEM reagents are generally considered to contain strong activators resulting in a rapid thrombin burst [12], the tissue factor concentration in the lyophilized reagents is unknown and may explain the higher variability of tissue factor activated parameters in canine blood samples.

The judgment of the variability depends on the classification of the CoV, for which we followed the approach of previous studies [7,30,31]. With Ex-TEM and Fib-TEM S tests, the parameters CT, CFT and ML should be interpreted with caution. Current viscoelastic testing guidelines do not suggest the necessity for duplicate measurements; however, due to the inter-duplicate variation, we suggest running duplicate measurements of (abnormal) Ex-TEM and Fib-TEM S tests to detect possible outliers and to create an overall larger database of values for further studies. In-TEM S, on the other hand, consistently showed very stable and reliable results and may therefore be the preferred primary test for interpretation of coagulation status in dogs. The ICC supports the results of CoV measurements.

Parameter wise, we identified considerably higher variability and lower correlation for CT, CFT and ML compared to alpha angle, MCF, MCE and G. This is consistent with the findings of previous studies [7,29] but in contrast to other studies that report low variability <15% or good correlation in dogs [8] and cats [11,32]. The early phase of clot initiation reflected by CT is known to be mainly dependent on the activator and most sensitive to small differences regarding sample handling and choice of activator, which would explain the higher intrarater variability of CT and CFT. The later phases of clot development are mainly influenced by platelet number and function as well as fibrin polymerization and only a little by the activator chosen [7,12]. In this group of healthy patients, ML was often 0 or 1%, explaining the high variability and the need to not only look at CoVs but also at the clinical impact. Comparisons of data in our study were, with a few exceptions, all statistically significant. Since most of the measurements remained within the reference interval despite variation, clinical relevance can be debated. Nevertheless, assessed with G, a parameter used for global coagulation status, we noticed a change in coagulation status in 10% of blood analyses of the same blood samples, which could have led to the initiation of incorrect therapies.

This study has some limitations. Due to channel limitations, the sample size is small and not all measurements for the comparison of jugular versus saphenous blood samples were run in duplicates, decreasing the number of comparisons. We also had to exclude some paired jugular-saphenous samples because tests were not run on the same device and the device was shown to have a significant effect on results. Regarding sample size, most analyses were statistically significantly different, indicating enough power for the detection of significant changes in all parameters. An additional limitation could be the multiple pipetting from a tube. Zambruni et al. suggested that repeated sampling from one tube for duplicate measurements should be avoided to prevent artificial hypercoagulability [33]. Since we did not find any evidence of corresponding systematic deviations in our data, we do not consider this to be relevant in our case. Additionally, when performing duplicate measurement in clinical practice, the same tube will be used for both analyses. Lastly, the inclusion of exclusively healthy dogs must be mentioned as a possible limitation, as inclusion of more hypo- and hypercoagulable tracings may change results. It may be assumed that the influence on duplicate measurements and device comparison is negligible. However, further studies are needed that also look at the influence of blood sampling localization on ROTEM S parameters in diseased animals.

## 5. Conclusions

CT and CFT parameters of Ex-TEM S and Fib-TEM S tests, as well as ML parameters of all three tests, were associated with high CoVs, and low ICC and should be interpreted with caution or measured in duplicates. Apart from these parameters, moderate-to-low variability was investigated for ROTEM S parameters, and In-TEM S showed an excellent performance. We recommend the same device and the same blood collection site for serial measurements. Jugular blood sampling using a syringe may be preferred over a free-flowing lateral saphenous blood sampling technique, as clearly more parameters from jugular venipuncture were within the reference interval.

## Figures and Tables

**Table 1:** Comparison of jugular and venous ROTEM parameters in simultaneously measured blood samples.

Parameter		Jugular			Saphenous		Jugular-Saphenous	Intraclass Correlation Coefficient (ICC)				CoV	*t*-test
	Unit	*n*	Mean	SD	Mean	SD	Mean Difference *	ICC	Lower CI	Upper CI	*p*	%	*p*
Ex-TEM													
CT	s	36	53✓	31	41✓	21	21	0.290	−0.014	0.552	0.030	27	<0.001
CFT	s	36	433	783	234✓	123	219	0.198	−0.117	0.484	0.108	20	0.069
α	°	36	50✓	11	55✓	10	7	0.545	0.200	0.755	0.002	11	<0.001
MCF	mm	36	42✓	8	45✓	7	5	0.646	0.287	0.825	0.001	8	<0.001
MCE		36	74✓	23	85✓	24	13	0.712	0.359	0.865	<0.001	13	<0.001
G		36	3711✓	1162	4242✓	1191	675	0.712	0.351	0.866	0.001	13	<0.001
ML	%	35	4✓	2	4✓	2	1	0.290	−0.014	0.552	0.030	21	<0.001
In-TEM													
CT	s	18	202✓	20	188✓	17	26	0	−0.383	0.369	0.588	9	<0.001
CFT	s	18	127✓	48	132✓	43	22	0.814	0.574	0.926	<0.001	12	<0.001
α	°	18	67✓	7	66✓	6	4	0.789	0.527	0.915	<0.001	4	<0.001
MCF	mm	18	57✓	7	57✓	7	2	0.934	0.832	0.975	<0.001	2	<0.001
MCE		18	139✓	42	140✓	49	11	0.941	0.850	0.978	<0.001	5	0.001
G		18	6966✓	2114	7012✓	2430	539	0.941	0.850	0.978	<0.001	5	0.001
ML	%	18	0.2✓	0.4	0.2✓	0.6	0.2	0.361	−0.130	0.704	0.070	71	0.187
Fib-TEM													
CT	s	19	106✓	266	120	269	21	0.982	0.954	0.993	<0.001	18	0.066
MCF	mm	19	4✓	2	6✓	4	2	0.246	−0.189	0.613	0.133	21	0.027
MCE		19	5✓	2	6✓	5	2	0.168	−0.269	0.561	0.227	21	0.053
G		19	237✓	87	313✓	242	105	0.167	−0.267	0.558	0.228	20	0.054

✓ within the reference interval. CI—confidence interval; CT—clotting time; CFT—clot formation time; α—alpha-angle; MCF—maximum clot firmness; MCE—maximum clot elasticity (E = 100 × MCF/(100−MCF); G—calculated measure of total clot strength (G = 5000 × MCF/(100−MCF); ML—maximum lysis; Ex-TEM—tissue factor-activated temogram; In-TEM—elagic acid-activated temogram; Fib-TEM—tissue factor-activated temogram with thrombocyte inhibition. * average absolute difference between the saphenous and jugular measurement for each group.

**Table 2:** Change of coagulation status with samples measured simultaneously from 2 different sampling sites (jugular vs. saphenous), as duplicates and on 2 different devices.

Change of Coagulation Status Based on Collection Site			
TEST	Coagulation Status	V. Jugularis	V. Saphena
Ex-TEM	normocoagulable	33/36 (92%)	32/36 (89%)
	hypocoagulable	2/36 (5%)	1/36 (3%)
	hypercoagulable	1/36 (3%)	3/36 (8%)
In-TEM	normocoagulable	16/18 (89%)	15/18 (83%)
	hypocoagulable	2/18 (11%)	2/18 (11%)
	hypercoagulable	0/18 (0%)	1/18 (6%)
Fib-TEM	normocoagulable	18/19 (95%)	17/19 (89%)
	hypocoagulable	1/19 (5%)	1/19 (5%)
	hypercoagulable	0/19 (0%)	1/19 (5%)
**Change of coagulation status between duplicates**			
**TEST**	**Coagulation status**	**Measurement 1**	**Measurement 2**
Ex-TEM	normocoagulable	16/23 (70%)	15/23 (65%)
	hypocoagulable	6/23 (26%)	6/23 (26%)
	hypercoagulable	1/23 (4%)	2/23 (9%)
In-TEM	normocoagulable	13/17 (76%)	13/17 (76%)
	hypocoagulable	4/17 (24%)	4/17 (24%)
	hypercoagulable	0/17 (0%)	0/17 (0%)
Fib-TEM	normocoagulable	18/21 (86%)	17/21 (81%)
	hypocoagulable	2/21 (9%)	2/21 (10%)
	hypercoagulable	1/21 (5%)	2/21 (10%)
**Change in coagulation status between 2 devices**			
**TEST**	**Mean coagulation status**	**Device 1**	**Device 2**
Ex-TEM	normocoagulable	4/7 (57%)	3/7 (43%)
	hypocoagulable	3/7 (43%)	4/7 (57%)
	hypercoagulable	0/7 (0%)	0/7 (0%)
In-TEM	normocoagulable	4/6 (67%)	4/6 (67%)
	hypocoagulable	2/6 (33%)	2/6 (33%)
	hypercoagulable	0/6 (0%)	0/6 (0%)
FibTEM-	normocoagulable	4/4 (100%)	3/4 (75%)
	hypocoagulable	0/4 (0%)	1/4 (25%)
	hypercoagulable	0/4 (0%)	0/4 (0%)

Ex-TEM—tissue factor-activated temogram; In-TEM—elagic acid-activated temogram; Fib-TEM—tissue factor-activated temogram with thrombocyte inhibition.

**Table 3:** Comparison of paired simultaneous ROTEM measurements analyzed by the same operator on the same device/Investigation of intra-operator/intrarater variation.

Parameter		Measurement 1			Measurement 2		Measurement 1–Measurement 2	Intraclass Correlation Coefficient (ICC)				CoV	*t*-test
	Unit	*n*	Mean	SD	Mean	SD	Mean Difference *	ICC	Lower CI	Upper CI	*p*	%	*p*
Ex-TEM													
CT	s	23	79✓	56	50✓	23	39	0.098	−0.229	0.445	0.290	34	0.001
CFT	s	23	558	713	616	759	429	0.225	−0.214	0.582	0.153	32	0.018
α	°	23	45✓	12	43✓	14	7	0.674	0.378	0.847	<0.001	13	<0.001
MCF	mm	23	37✓	10	37✓	11	6	0.711	0.427	0.867	<0.001	13	<0.001
MCE		23	62✓	27	63✓	31	15	0.801	0.586	0.911	<0.001	19	<0.001
G		23	3124✓	1328	3164✓	1552	733	0.801	0.585	0.911	<0.001	19	<0.001
ML	%	22	3✓	2	3✓	2	1	0.761	0.513	0.893	<0.001	40	<0.001
In-TEM													
CT	s	18	189✓	35	179✓	20	14	0.779	0.433	0.916	<0.001	5	<0.001
CFT	s	18	139✓	56	129✓	40	23	0.787	0.525	0.914	<0.001	12	<0.001
α	°	18	66✓	8	67✓	6	4	0.793	0.538	0.917	<0.001	4	<0.001
MCF	mm	17	55✓	6	56✓	6	1	0.970	0.917	0.989	<0.001	1	0.001
MCE		17	129✓	35	133✓	34	6	0.973	0.927	0.990	<0.001	3	<0.001
G		17	6437✓	1768	6662✓	1676	302	0.973	0.928	0.990	<0.001	3	<0.001
ML	%	16	0.1✓	0.2	0.1✓	0.5	0.2	0	−0.566	0.453	0.581	141	0.188
Fib-TEM													
CT	s	21	107✓	258	212	573	111.95	0.720	0.436	0.875	<0.001	21	0.128
MCF	mm	21	5✓	2	5✓	2	1	0.870	0.706	0.945	<0.001	12	0.008
MCE		21	5✓	2	5✓	3	1	0.861	0.690	0.941	<0.001	12	0.006
G		21	237✓	113	246✓	137	37	0.870	0.709	0.945	<0.001	13	0.004

✓ within the reference interval. CI—confidence interval; CT—clotting time; CFT—clot formation time; αalpha-angle; MCF—maximum clot firmness; MCE—maximum clot elasticity (E = 100 × MCF/(100−MCF); G—calculated measure of total clot strength (G = 5000 × MCF/(100−MCF); ML—maximum lysis; Ex-TEM—tissue factor-activated temogram; In-TEM—elagic acid-activated temogram; Fib-TEM—tissue factor-activated temogram with thrombocyte inhibition. * average absolute difference between the first and second measurement for each group.

**Table 4:** Comparison of paired simultaneous ROTEM measurements analyzed by the same operator on two different devices/investigation of in-between device variation.

Parameter		Device 1			Device 2			Device 1–Device 2	Intraclass Correlation Coefficient (ICC)				CoV	*t*-test
	Unit	*n* Duplicate Measurments	Δ Mean	SD	*n* Duplicate Measurments	Δ Mean	SD	Mean Difference *	ICC	Lower CI	Upper CI	*p*	*%*	*p*
Ex-TEM														
CT	s	7	88	38	7	68✓	26	34	0.132	−0.535	0.754	0.362	33	0.031
CFT	s	7	648	297	7	944	890	656	0	−0.772	0.711	0.511	46	0.048
α	°	7	40	8	7	38	11	7	0.565	−0.299	0.911	0.081	15	0.026
MCF	mm	7	32✓	6	7	32✓	8	5	0.710	−0.059	0.945	0.032	12	<0.001
MCE		7	48✓	14	7	50✓	20	10	0.782	0.139	0.959	0.014	16	0.001
G		7	2415✓	713	7	2498✓	990	518	0.784	0.149	0.960	0.013	16	<0.001
ML	%	7	3	1	7	3	2	1	0.676	0.030	0.934	0.021	45	<0.001
In-TEM														
CT	s	6	192✓	28	6	187✓	32	7	0.956	0.744	0.994	<0.001	2	0.057
CFT	sec	6	143✓	33	6	138✓	26	14	0.877	0.412	0.982	0.003	8	0.001
α	°	6	64✓	5	6	66✓	4	3	0.789	0.170	0.967	0.011	3	0.002
MCF	mm	5	56✓	7	6	53✓	4	3	0.698	0.011	0.949	0.024	3	0.154
MCE		5	136✓	51	6	117✓	17	20	0.472	−0.300	0.900	0.110	8	0.255
G		5	6826✓	2581	6	5831✓	837	1020	0.469	−0.302	0.899	0.111	9	0.249
ML	%	4	0✓	0	6	0.1✓	0.2	0.1	0.000	−0.755	0.755	0.500	141	0.363
Fib-TEM														
CT	s	4	40✓	8	4	226	361	187	0.040	−0.819	0.884	0.471	45	0.366
MCF	mm	4	4✓	1	4	3✓	1	1	0.500	−0.606	0.958	0.170	23	0.016
MCE		4	4✓	1	4	3✓	1	1	0.572	−0.522	0.965	0.130	24	0.016
G		4	188✓	51	4	160✓	63	39	0.761	−0.069	0.982	0.034	19	0.016

✓ within the reference interval. CI—confidence interval; CT—clotting time; CFT—clot formation time; α—alpha-angle; MCF—maximum clot firmness; MCE—maximum clot elasticity (E = 100 × MCF/(100−MCF); G—calculated measure of total clot strength (G = 5000 × MCF/(100−MCF); ML—maximum lysis; Ex-TEM—tissue factor-activated temogram; In-TEM—elagic acid-activated temogram; Fib-TEM—tissue factor-activated temogram with thrombocyte inhibition; Δ Mean—average absolute difference between the respective first and second measurement for each device. * average absolute difference between device 1 and 2.

## Data Availability

Data are available on request.
